# Bone marrow‐derived extracellular vesicles modulate the abundance of infiltrating immune cells in the brain and exert an antiviral effect against the Japanese encephalitis virus

**DOI:** 10.1096/fba.2022-00071

**Published:** 2022-10-28

**Authors:** Naina Soni, Aarti Tripathi, Sriparna Mukherjee, Suchi Gupta, Sujata Mohanty, Anirban Basu, Arup Banerjee

**Affiliations:** ^1^ Laboratory of Virology Regional Centre for Biotechnology Faridabad India; ^2^ National Brain Research Centre Manesar India; ^3^ DBT‐Centre of Excellence for Stem Cell Research, Stem Cell Facility All India Institute of Medical Sciences New Delhi India; ^4^ Department of Pharmacology and Physiology Pavilion Roger‐Gaudry, Universite de Montréal Montréal Québec Canada

**Keywords:** antiviral genes, EVs, JEV, MSC, Neurospheres

## Abstract

Mesenchymal stem cells (MSCs) have regenerative capacity and have reported a beneficial effect on the Japanese encephalitis virus (JEV) in an encephalitis model. However, the MSCs do not cross the blood–brain barrier and have other disadvantages limiting their therapeutic utility scope. Recently, there has been a shift in concept from a cell‐based to a cell‐free approach using MSCs‐derived extracellular vesicles (MSC‐EVs). The MSC‐EVs retain regenerative and immunomodulatory capacity as their parental cells. However, the role of MSC‐EVs in limiting JEV pathology remains elusive. In this study, we have used Bone marrow (BM)‐derived EV (BM‐EVs) and assessed their effect on JEV replication and pathogenesis in primary neuronal stem cells and a murine model. The in vitro and in vivo studies suggested that BM‐derived EVs delay JEV‐induced symptoms and death in mice, improve the length of survival, accelerate neurogenesis in primary neuronal stem cells, reduce JEV‐induced neuronal death, and attenuate viral replication. BM‐EVs treatment upregulated interferon‐stimulated genes. Flow cytometry analysis revealed a reduction in the frequency of macrophages. At the same time, CD4+ T cells and neutrophils were significantly augmented, accompanied by the alteration of cytokine expression with the administration of BM‐EVs, reinforcing the immunomodulatory role of EVs during JEV‐induced encephalitis. In conclusion, our study describes the beneficial role of BM‐EVs in limiting JEV pathology by attenuating virus replication, enhancing antiviral response, and neurogenesis in primary neuronal stem cells. However, BM‐EVs do not seem to protect BBB integrity and alter immune cell infiltration into the treated brain.

## 1 INTRODUCTION

Japanese encephalitis virus (JEV) is a single‐stranded, linear, positive‐sense RNA (~11 kb in genome length) virus belonging to the family *Flaviviridae*. This virus can infect brain resident cells and is the leading cause of viral encephalitis in Asia and India. About 70,000 cases are reported yearly, and the mortality rate is as high as 20%–30%.^1^ This virus primarily affects children and adults, and survivors (30%–50%) suffer long‐term neurological sequels (http://nvbdcp.gov.in/je‐new.html). JEV attenuated viral vaccine (SA‐14‐14) is widely available with varying efficacy. Currently, no effective anti‐JEV drugs or methods are available to treat JEV‐induced neuroinflammation.^2^ Only life‐sustaining treatment is provided to patients. Therefore, there is an unmet need to research an alternative approach to treating JEV patients.

The stem cells, including mesenchymal stem cells (MSCs) and neural/progenitor stem cells (NPCs), transplantation approach finds substantial beneficial effect in experimental CNS disease models.^3^, ^4^, ^5^ In this context, adult stem cells (e.g., mesenchymal stem cells, MSCs) hold great promise as they have immune regulatory and tissue repair properties; they are easily expandable in vitro culture conditions with a lower risk of immune rejection.^6^ Furthermore, under proper in vitro conditions, these cells can be differentiated into neuronal cells.^7^, ^8^, ^9^ It is now suggested by a recent study that MSCs decreased JEV replication through the expression of IFN‐β and IFN‐α.^10^ MSCs can control local inflammation and maintain tissue homeostasis. MSCs can also regulate the BBB integrity and function of microglia.^11^, ^12^ Collectively, all evidence indicates that MSCs hold great potential in JEV‐induced consequences.

However, the cells do not cross the blood–brain barrier (BBB) and have many disadvantages,^13^ thus limiting their scope for therapeutic utility. It is thought that these cells secrete immune‐modulatory or neurotrophic paracrine factors that help in resolving inflammation.^14^ This paracrine hypothesis has inspired an alternative outlook on using stem cells in regenerative neurology.^15^, ^16^, ^17^, ^18^ In this context, the injured brain's repair may be achieved by injecting the biologics secreted through the small vesicles called extracellular vesicles (EVs) by stem cells rather than implanting stem cells themselves for direct cell replacement.^19^

EVs are nanoscale vesicles that contain various biomolecules, including proteins, RNA, and lipids from their parental cell. Unlike cells, EVs can cross BBB and are replication incompetent. Therefore, EVs have gained importance in regenerative medicine, especially for treating neurological disorders.^20^, ^21^, ^22^

MSC‐derived EVs can recapitulate the biological activity of MSCs and may serve as an alternative to whole‐cell therapy. EVs may present considerable advantages over their cellular counterparts due to a higher safety profile, lower immunogenicity, and the inability to directly form tumors.^23^ In contrast to the relatively large MSCs (30–60 μm in diameter), nano‐sized EVs have the potential to migrate efficiently to the target organ after infusion without getting trapped in the lung microvasculature.^24^ MSC‐derived EVs participate in intercellular communication and carry proteins, mRNA, and microRNA (miRNA, into targeted cells).^25^

However, it is unknown whether MSCs‐derived EVs can attenuate the encephalitis caused by JEV. This study has explored the efficacy of EVs derived from Bone Marrow (BM) of MSCs on suppressing JEV replication in neuronal stem cells. In vitro study suggested that BM‐derived EVs were attenuating viral replication. We further extended our study with BM‐derived EVs to explore their capacity for neuronal differentiation, neuronal protection, and peripheral immune cell infiltration in JEV infected in primary neuronal stem cells and mice brains.

## 2 MATERIALS AND METHODS

### 2.1 Animals

Experiments were performed in C57BL/6 mice. Mice were housed in a 12:12 light: dark cycle with constant temperature and humidity, ad libitum access to food and water at small animal facility, RCB, Faridabad. All procedures followed the guidelines of the Committee for Control and Supervision of Experiments on Animals, Ministry of Environment and Forestry, Government of India, and approved by the Regional Centre for Biotechnology ethics committee Approval no RCB/IAEC/2019/050.

The stem cell isolation, culture, and exosome isolation were initiated only after approval from Institutional Committee for Stem Cell Research (ICSCR), All India Institute of Medical Sciences, New Delhi, India, Reference No. IC‐SCR/55/16(R). All the samples were obtained after taking the donors' consent according to the guidelines of the ethical committee.

### 2.2 C17.2 Cell

The C17. 2 cells are mouse‐derived multipotent neural stem cells isolated from the cerebellum and immortalized by avian myelocytomatosis viral‐related oncogene (v‐myc) transfection.^26^, ^27^ The C17.2 Cell line was a kind gift from Prof. Anirban Basu, NBRC. C17.2 cells were grown in Dulbecco's Modified Eagle high glucose Medium (DMEM, Gibco Carlsbad) supplemented with 10% FBS (Fetal Bovine Serum), 5% Horse Serum (Gibco Carlsbad), and 1% antibiotics as an adherent culture.

### 2.3 Virus generation and propagation and Virus titration by plaque‐forming assay

The cell culture grew Japanese encephalitis virus (P20778 strain) is propagated in C6/36 mosquito cell line and used for in vitro experiments. Whereas JEV‐S3 (P20778 strain) adapted in adult mice (JEV‐S3) was used for in vivo study as described earlier^28^ and titration was done in Vero cells by plaque assay. For in vivo virus generation, C57BL/6 were injected with 1 × 10^7^ pfu of JEV virus and monitored until they developed encephalitis symptoms. Brains were harvested and homogenized in serum‐free minimum essential medium (MEM) (Invitrogen) and centrifuged at 10,000 × g for 15 min. The supernatant was filtered through a 0.22‐μm filter, titrated in Vero cells by plaque assay as described earlier.^28^ Plaques were counted, and the virus titer was calculated using the following formula: virus titer (pfu/ml) = average pfu/volume of infection (ml) dilution factor.

### 2.4 Mesenchymal stem cell isolation and exosome purification

Mesenchymal stem cell isolation and exosome purification were performed at the Stem Cell Facility, All India Institute of Medical Sciences (AIIMS), New Delhi, using a previously standardized protocol.^29^, ^30^ Briefly, bone marrows were collected from the donors (*n* = 3) undergoing the routine medical test procedure at AIIMS, New Delhi. The MSCs were isolated and cultured as described previously by our group.^30^ Briefly, MSCs were passaged and identified by surface marker analysis and in vitro differentiation assays.^31^ MSCs in passages 3–5 from three subjects were used for the experiments below.

The serum‐free conditioned media from MSCs cultured for 48 h was collected and exercised for EV isolation. The cellular debris was removed by centrifugation at 300× *g* for 10 min, then centrifugation at 10,000× *g* for 30 min to remove apoptotic bodies and other micro‐vesicles. The conditioned media were loaded slowly over 30% sucrose solution, forming a layer, and ultra‐centrifuged at 100,000× *g* at 4°C for 90 min. The supernatant was discarded, and the sucrose layer was resuspended in 1X PBS and ultra‐ centrifuged again at 100,000× *g* at 4°C for 90 min. Lastly, the pellet was resuspended in 1X PBS. The protein concentration of EVs was measured by Micro BCA Protein Assay Kit (#23235, Thermo Fisher).

### 2.5 Neurospheres isolation, culture, and treatment

Neurospheres were isolated from the subventricular zone of 7‐day‐old mouse as described earlier.^32^ Briefly, the subventricular zone was dissected and digested enzymatically with papain enzyme at 2 mg/ml concentration and DNase I. Subsequent washes were given with the FBS‐supplemented media. Finally, cells were seeded in DMEM‐F12, supplemented with B27 supplements, 50 μg/ml gentamycin (Gibco), and growth factors Epidermal Growth Factor (20 mg/ml) and basic Fibroblast Growth Factor (10 mg/ml) (R&D Systems). Cultured cells were treated with three different concentrations: 10 μg, 20 μg, 30 μg/ml of EV equivalent protein from BM‐MSCs, and observed for 7 days.

### 2.6 MTT assay

C17.2 cells were plated on 96‐well plates at a density of 1 × 10^4^ cells per well to check the viability. Cells were incubated with five different concentrations of BM‐EVs for 24 h. Cell Titer 96 Aqueous one solution reagent (20 μ; Cell Titer 96® Non‐Radioactive Cell Proliferation Assay; Promega) was added to each well 4 h before the harvesting of the cells. Absorbance was measured at 570 nm using a 96‐well multimode plate reader (Synergy 2Multi‐Mode Reader; BioteK).

### 2.7 Japanese encephalitis virus infection in vitro and in vivo

Cells were mock or infected with JEV at MOI = 5 for 24 h and treated with three different concentrations 10 μg, 20 μg, and 30 μg/ml of EV equivalent protein. Cells were then processed for immunostaining, qRT‐PCR, and Western blot.

In an in vivo model system, C57BL/6 mice of age P21 were injected with 1 × 10^7^ plaque‐forming unit of JEV through the intra‐peritoneal (IP) route. Following 12 h postinfection, mice were administered 50 μg of protein equivalent EVs twice a day (i.e., a total of 100 μg of BM‐EVs, which was equivalent to 3.0 X 10^9^ ± 1.8 X 10^7^ particles/ ml as estimated by Nano Sight) through the intraperitoneal route in C57BL/6 mice for 7 days. In another set of experiments, P10‐old mice received 20 μg of BM‐EVs through the intracranial route on day1 and 3.

### 2.8 In vitro paracrine assay

C17.2 cells, either mock‐ or JEV‐infected, were incubated with conditioned media (CM) harvested from human bone marrow, Adipose tissue‐derived cultured MSCs for 24 h. After incubation, cells were processed for qRT‐PCR.

### 2.9 Bone marrow‐derived extracellular vesicles staining and uptake assay

BM‐EVs were incubated with PKH67 dyes (Cat# MINI67 Sigma‐Aldrich), and unbound excess dyes were removed as per manufacturer protocols. Neurospheres and C17.2 cells were incubated with either unstained BM‐EVs or BM‐EVs stained with PKH67 dye and incubated for different time points. After incubation, the green fluorescent labeled BM‐EVs, and cell nucleus (blue) were captured using a confocal microscope as well as by flow cytometry at different time points.

### 2.10 RNA isolation and qRT‐PCR

RNA was extracted from the brain, cells, and neurospheres by using the RNeasy kit (Qiagen) with in‐column DNase digestion formula and quantified using Nanodrop. cDNA was prepared from 200 ng of RNA in two steps using the GoScript reverse transcription system (Promega). The primers used for this study were published previously.^32^ All PCR reactions were performed in triplicate by the real‐time fluorescence detection method by using SYBR green DNA‐binding fluorescent dye (SYBR Premix *Ex Taq*; TaKaRa) on a QuantStudio 6 Flex real‐time PCR (Applied Biosystems) detection instrument. Relative expression was calculated using the threshold cycle (*CT*), and *Gapdh* (glyceraldehyde‐3‐phosphate dehydrogenase) was used as an internal control.

### 2.11 Immunocytochemistry

C17.2 cells were grown on Poly‐L‐lysine‐coated coverslips and mock or JEV infected. Cells were fixed with 4% paraformaldehyde and permeabilized with 0.25 triton X‐100 for 15 min at RT. Blocking is done with 5% Bovine serum albumin (BSA; Sigma, A7906) in PBS for 1 h at RT. Further incubated with Primary antibody JEV‐ NS1(1:1000, in‐house) and Nestin (1:100, Merck Millipore MAB353, RRID:AB_94911) for 12 h. Cells were washed thrice with PBS and then incubated with secondary antibodies (goat anti‐rabbit conjugated with Alexa flour 488; Invitrogen and goat anti‐mouse conjugated with Alexa flour 564; Invitrogen) diluted 1:500 with PBS for 1 h at RT in the dark. After incubation, cells were washed thrice with PBS, and coverslips were mounted with a prolonged anti‐fade reagent (P36935, Invitrogen) with DAPI. Images were captured on an Olympus FV3000 confocal microscope with a 60X (NA 1.4) objective.

### 2.12 Japanese encephalitis virus replication study in C17.2 cells and Neurospheres

Cells were seeded at a density of 1.5 × 10^5^ cells in a 12‐well plate and infected with JEV at MOI = 5 for 1 h in serum‐free media. The cells were then incubated with three different concentrations (10, 20, and 30 μg) per ml of EVs for three different time points (12, 24, 36 h). The cells were harvested to check the viral RNA level by qRT‐PCR, as described earlier.^33^ Reverse transcription was performed using the cDNA synthesis kit (GoScript reverse transcription system [Promega]). The primers used for JEV amplification were (forward) 5′ AGAGCACCAAGGGAATGAAATAGT 3′; (reverse) 5′ AATAAGTTGTAGTTGGGCACTCTG 3′. These primers can detect both sense and anti‐sense strands of the viral genome.

### 2.13 Brain histology and immunohistochemistry

Mice were anesthetized with ketamine and transcardially perfused with cold PBS, followed by 4% paraformaldehyde. Brains were extracted and stored in 4% paraformaldehyde (Sigma‐Aldrich) for 24 h for fixation and then left in 30% sucrose until immersion. Brains were then washed with PBS, embedded in OCT (Tissue Tek), and stored at −20°C until further use. Sections (12 μm) of the brain were made with the help of a cryostat (Microm HM550, Thermo Fisher Scientific) and mounted on slides. For Hematoxylin‐Eosin staining, sections were washed twice with PBS and stained with Hematoxylin (Sigma‐Aldrich) followed by incubation in 0.5% HCl. Brain sections were washed and treated with a gradient of alcohol (30%, 50%, and 70%) before staining with Eosin (Sigma‐Aldrich). The sections were then treated with a gradient of alcohol (90% and 100%), rinsed with xylene, and mounted with DPX (Sigma‐Aldrich). They were then examined with an Eclipse Ti (Nikon) imaging system. Images were captured at 20× and 40× magnification and analyzed using NIS‐Element AR 4.6 software.

### 2.14 Immunostaining

Mice were perfused with sterile, cold 1X PBS followed by 4% paraformaldehyde (PFA). The brains were removed and kept in 4% paraformaldehyde for 24 h at 4°C, followed by incubation in 30% sucrose solution until immersion. Brains were taken out and embedded in OCT (Tissue‐Tek) and frozen at −20°C. Twelve‐micrometer cryostat sections of the brain were made with the help of cryostat (Microm HM550; Thermo Scientific) and mounted on slides. The antigen retrieval process was performed at 95°C using antigen unmasking solution (Vector Laboratories). After permeabilization with 0.1% Triton X‐100 and blocking with 10% bovine serum, the sections were incubated overnight with a primary antibody against antigens. The primary antibodies used were as follows: in‐house rabbit polyclonal antibody against JEV NS1 protein (1:1000), mouse anti‐JEV E glycoprotein (1:20; ab41671, Abcam, RRID:AB_775814), mouse anti‐IBA1 antibody (1:100; MABN92, EMD Millipore, Darmstadt, Germany, RRID:AB_10917271), mouse anti‐GFAP (1:50; MAB3402, EMD Millipore, RRID:AB_94844), rabbit anti‐NeuN (1:100; ABN78, EMD Millipore, RRID:AB_10920751) and rabbit anti‐cleaved caspase‐3 (1:50; 9661S, Cell Signaling Technology, Beverly, MA, USA, RRID:AB_2341188). After washing with PBS, the sections were incubated for 1 h with fluorochrome‐conjugated secondary antibodies: Alexa Fluor 568 goat anti‐rabbit (1:500; A11011, Invitrogen, RRID:AB_143157), Alexa Fluor 488, goat anti‐mouse (1:500; A11029, Invitrogen, RRID:AB_2534088), Alexa Fluor 568 goat anti‐mouse (1:500; A11004, Invitrogen, RRID:AB_2534072), and Alexa Fluor 488, goat anti‐rabbit (1:500; A11008, Invitrogen, RRID:AB_143165). Following a 1 h wash with PBS, 4′, 6‐diamidine‐2′‐ pheynylindole dihydrochloride (DAPI; Invitrogen) was used to stain the nuclear DNA. Images were acquired at 60X magnification using a confocal microscope (Olympus, FV 1000) and analyzed with FluoView (FV 31S‐SW) software.

### 2.15 Immune cell isolation from the brain

Mice were anesthetized with ketamine at a dose of 87 mg/kg body weight, followed by perfusion with ice‐cold PBS. Brains were harvested and homogenized with a Dounce homogenizer in HBSS buffer. Homogenate was transferred to prepare 30% isotonic Percoll, which was then over‐layered on 70% isotonic percoll (Sigma‐Aldrich). The gradient was centrifuged at 500 g for 30 min at 18°C. Mononuclear cells were collected from the 30/70 interface and washed twice with PBS. Collected cells were then stained for macrophages, monocytes, and T cells. Cells were first incubated with anti‐mouse CD16/32 antibody TruStain FcX as per manufacturer's protocol (BioLegend) for 15 min at 4°C to block Fc receptors and then simultaneously stained with fluorochrome‐conjugated antibodies (APC‐CD11b, FITC‐CD45 [at dilution 1:100; Miltenyi Biotec, Gladbach, Germany, RRID:AB_2819369; RRID:AB_2727575], FITC‐ Ly6C [at dilution 1:50; BioLegend Cat# 128005,RRID:AB_1186134], V450‐Ly6G [at dilution 1:50; BioLegend RRID:AB_2637124]). For T cell immune profiling, all of the antibodies were procured from BioLegend (BV510 anti‐mouse CD3ε [at dilution 1.5:50, RRID:AB_1240509], PerCP/Cyanine5.5 anti‐mouse CD4 [at dilution 1:50, RRID:AB_893324], Brilliant Violet 421 anti‐mouse CD8a [at dilution 1:50, RRID:AB_2814057], and APC/Cyanine7 anti‐mouse NK‐1.1 [at dilution 1:25], RRID:AB_830871). Cells were then rinsed with FACS buffer and run on the BD FACS Verse (BD Biosciences, San Jose, CA). Cells were fixed and permeabilized for intracellular staining using BD Cytofix/Cytoperm (BD Biosciences) as per the manufacturer's protocol. Data were analyzed using BD FACS Suite v1.0.6 (BD Biosciences) and FlowJo v10 (FlowJo LLC).

### 2.16 Cytokine bead array

Cytokine bead array (CBA) was performed quantitatively to measure the cytokine level. Eight cytokines (IL4, IL10, IFNγ, IL17, IL2, IL6, IL13, and TNFα) were measured using an assay kit procured from BioLegend, and data were analyzed using CBA software (FCAP 3.0.1 and Qognit). Thirty micrograms of protein lysate were used for analysis. Data were acquired using CellQuest Pro software in the FACS Calibur cell analyzer (BD Biosciences). The quantity of the cytokines detected in the lysate samples was measured against the standard curve obtained from the defined concentration of protein provided with the assay kit.

### 2.17 Statistical analysis

Results from all experiments were performed in triplicate, and statistical significance was performed using paired two‐tailed Student's *t*‐test using GraphPad Prism software (version. 8. 3.1, GraphPad Software, Inc.) Differences with *p* < 0.05 were considered significant.

## 3 RESULTS

### 3.1 Differential effect of mesenchymal stem cells secretomes on Japanese encephalitis virus replication in neuronal stem cells

**FIGURE 1:**
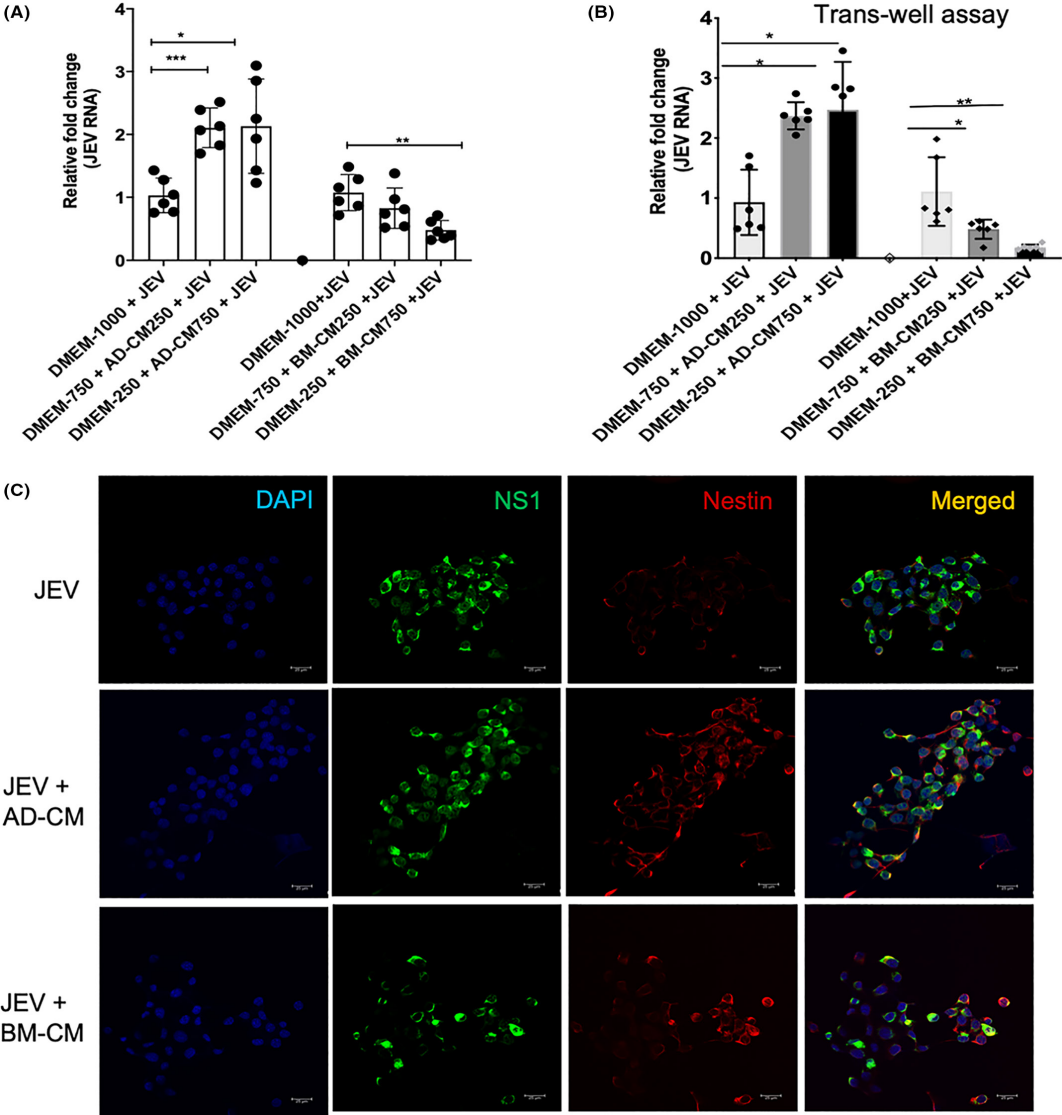
Effect of conditioned media from different Mesenchymal stem cell sources and their paracrine effect on JEV. (A) C17.2 cells were infected with JEV at MOI = 5 and incubated with conditioned media at two concentrations, 250 and 750 μl, for 24 h. Cells were lysed, and the viral load was measured using RT‐PCR. (B) Trans‐well assay was performed in 12‐well trans plates of pore size 0.4 μm. C17.2 cells were seeded in the upper compartment of the trans‐well and infected with JEV at MOI 5 for 1 h. Conditioned media (CM) of two different concentrations from all three sources was poured into the lower compartment of the trans well plate. After 24 h incubation, cells were harvested and processed for RT‐PCR to check the viral load. All qRT‐PCR data are represented as mean ± SD, *p < 0.05, **p < 0.001. (C) Cells (C17.2) were infected at MOI = 5 and incubated with 750 μl (BM‐CM or AD‐CM) + 250 μl of DMEM for 24 h. Immunofluorescence was performed to visualize the virus infectivity posttreatment in cells. The virus was marked using NS1 (green), Nestin (red) stem cell marker, and the nucleus was stained using DAPI (blue). AD, Adipose tissue; BM, Bone marrow; CM, Conditioned media.

MSCs can exert their effect in a paracrine manner by releasing bioactive molecules. To evaluate the impact of MSC paracrine signaling on JEV infection, we replaced the culture medium of JEV‐infected C17.2 cells with the MSCs' conditioned medium (CM). In this study, we used MSCs conditioned media obtained from two different sources, Bone marrow (BM) and Adipose tissue (AD). The results showed that the JEV infection rate of BM conditioned medium‐treated C17.2 cells was significantly reduced in a dose‐dependent manner compared with the cells treated with conditioned medium from AD cells (Figure 1A). To further verify this finding, we performed a trans‐well‐based assay to evaluate the paracrine function of MSCs. The secreted components of MSCs placed in the lower chamber passed through the membrane to the upper chamber seeded with C17.2 cells (Figure 1B). The results showed that JEV infection on C17.2 cells was primarily suppressed by coculture with BM‐MSCs in a dose‐dependent manner. The CM from AD cells did not exert any anti‐JEV activity; instead, it increased JEV replication (Figure 1B). The inhibitory effect of trans‐well‐based coculture was consistent with the effect observed with a conditioned medium. Still, it was even more potent, probably because of the persistent MSCs' paracrine signaling. Similar experiments were performed in C17.2 cells, where cells were fixed and stained for viral NS1 proteins (Green) and NeuN protein (Red). DAPI was used to stain the nucleus. The results showed that JEV infection on C17.2 cells was primarily suppressed by coculture with BM‐CM (Figure 1C), suggesting that BM‐CM exerts an inhibitory effect on JEV replication via paracrine.

Thus, our results suggested that BM MSC‐secreted components were capable of eliciting robust anti‐JEV responses.

### 3.2 Bone marrow‐derived extracellular vesicles antiviral activity against Japanese encephalitis virus infection

**FIGURE 2:**
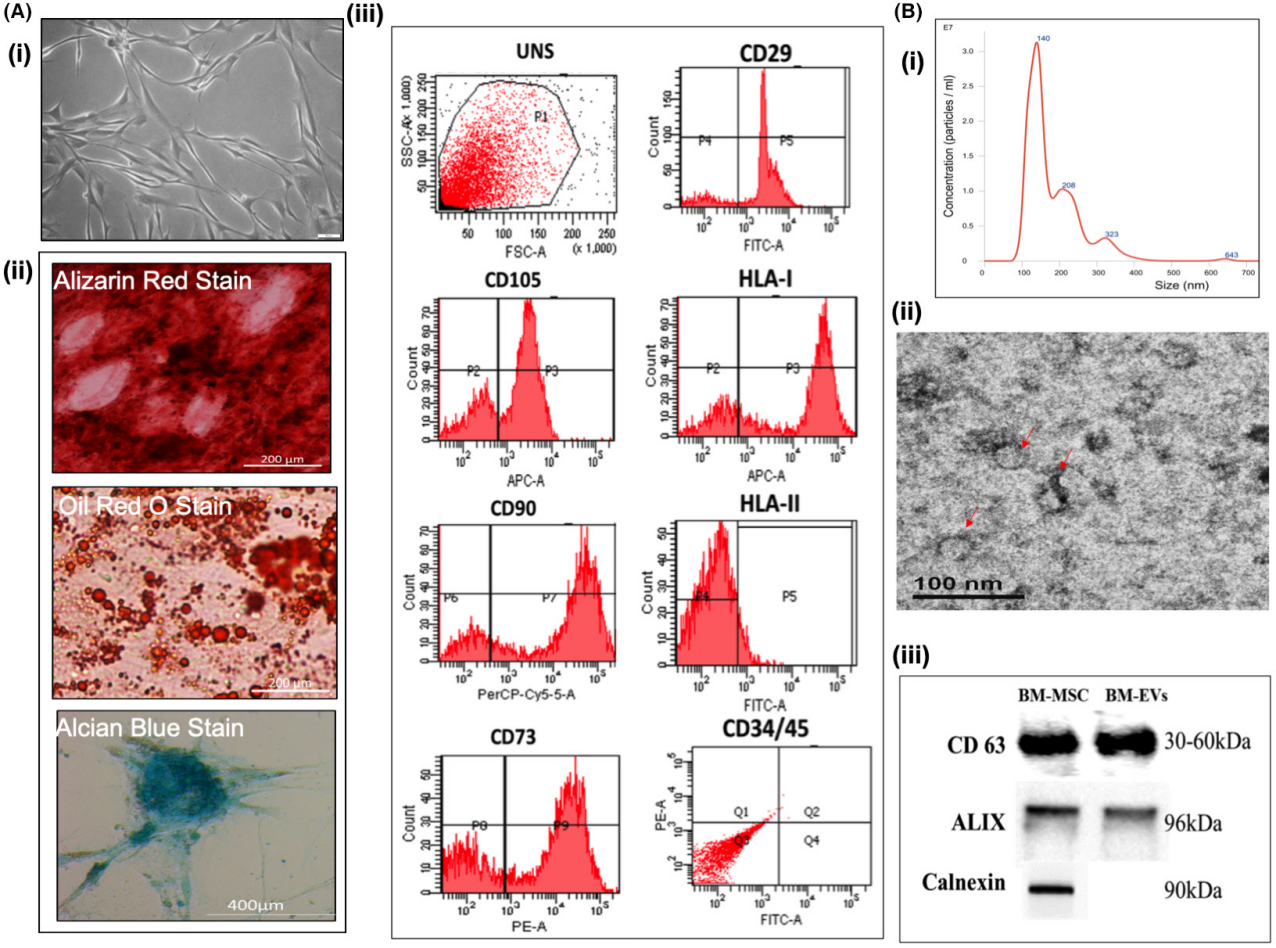
(A) Characterization of BM‐MSCs. (i) Phase‐contrast microscopy of MSCs (magnification 10×). (ii) Tri‐lineage differentiation potential of MSCs showing osteogenesis as determined by Alizarin red staining of the extracellular mineralized matrix, adipogenesis as determined by Oil red O staining of lipid droplets, and Chondrogenesis as determined by Alcian blue staining of proteoglycans. (iii) Surface marker profiling by Flow Cytometer. (B) Characterization of EVs (i) EVs size distribution analysis using nanoparticle tracking analysis (NTA). (ii) Morphological analysis of EVs by Transmission Electron Microscope (TEM). Red arrows were indicating cup‐shaped EVs (Bar = 100 nm; 29 KX) .(iii) Expression of EVs‐specific markers CD63 and ALIX by western blotting.

EVs play a vital role in MSC paracrine signaling. So, we investigated whether they were responsible for the anti‐JEV activity. Bone marrow‐derived MSCs were characterized by in vitro differentiation assays and surface marker analysis, as shown in Figure 2A (i–iii). The Phase‐contrast microscopy clearly revealed the spindle‐shaped morphology, which is the characteristic of tissue‐specific hMSCs. The tri‐lineage differentiation potential was evaluated by staining bone marrow‐derived MSCs with Alizarin red, Oil red O, and Alcian blue for “Osteocytes,” “Adipocytes,” and “Chondrocytes” differentiation, respectively (Figure 2A [ii]). The presence and absence of specific cell surface markers were evaluated by flow cytometry. As shown in Figure 2A (iii), Bone marrow‐derived MSCs were found positive for CD29, CD73, CD90, CD105, and HLA I and negative for HLA II and hematological markers CD34/45.

The extracellular vesicles (EVs) were purified from cell culture supernatant collected from passage 3–5 cells by one‐step ultracentrifugation methods as described earlier.^29^ The purified EVs were characterized based on the guidelines issued by the International Society for Extracellular Vesicles (ISEV).^34^ Nanoparticle tracking analysis (NTA) of purified exosomes revealed that the majority of BM‐EVs fall within the size range of small vesicles with a mean diameter of 126 nm (*n* = 3 donors) with a peak <200 nm (Figure 2B [i]). Transmission Electron Microscopy (TEM) was used to see the morphology. The BM‐EVs were found to be both round and cup‐shaped vesicles, corresponding to the size of exosomes and maintaining their integrity (Figure 2B [ii]).

In the Western blots, characteristic bands for CD63 and Alix were also detectable. The absence of the calnexin band in BM‐EVs but not in its parental cells further confirmed that the isolated small extracellular vesicles are pure exosomes and not contaminating vesicles from other compartments of the cell (Figure 2B [iii]).

**FIGURE 3:**
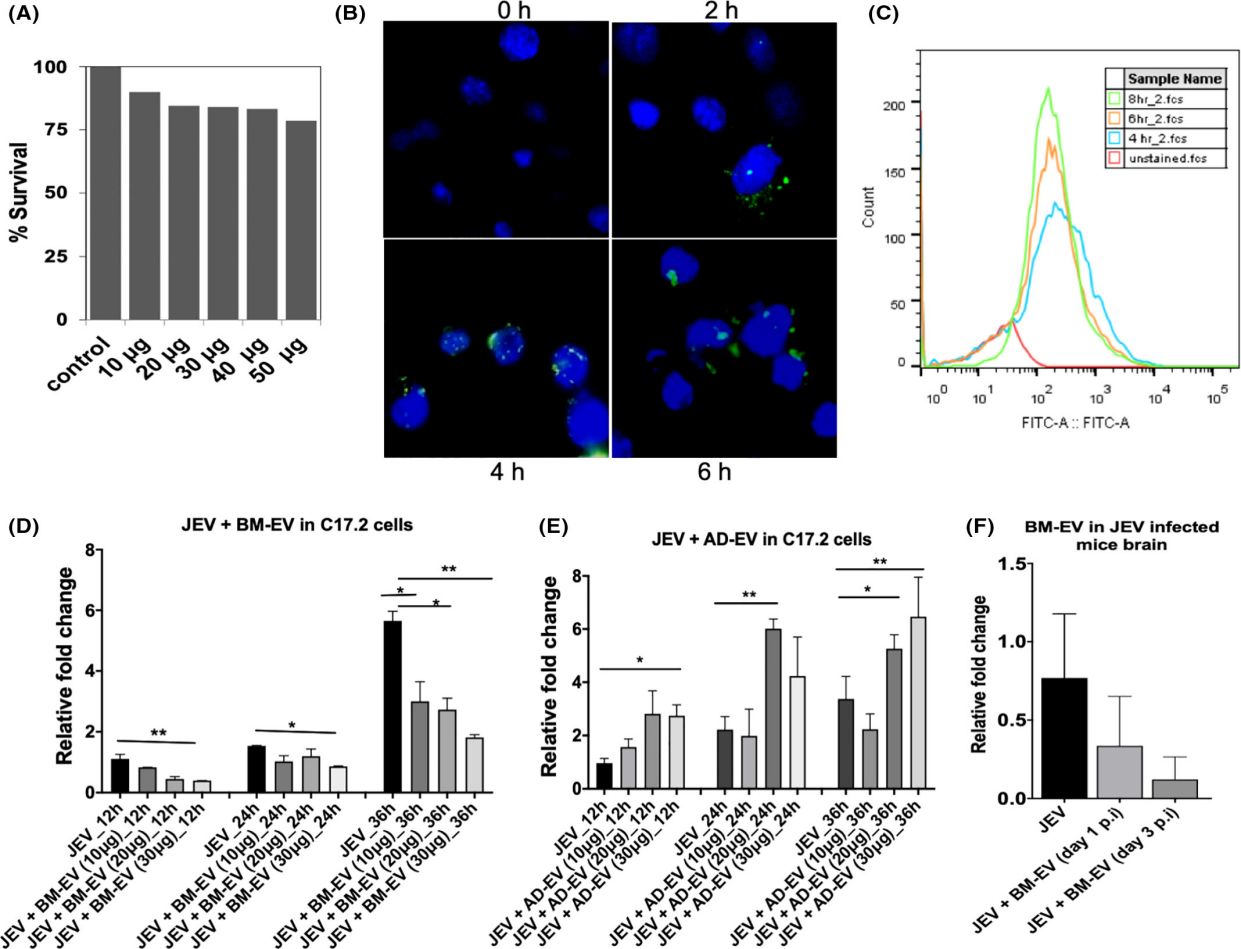
Effect of bone marrow‐mesenchymal stem cells (BM‐MSCs) derived conditioned media (CM) and EVs on JEV replication. (A) MTT assay was performed to check the cell viability at different concentrations of EVs. (B) Cells were incubated with PKH67‐labeled EVs (green) and visualized under the microscope at indicated time points. DAPI (blue) was used to stain the nucleus. (C) Flow cytometry analysis showed an increase in green fluorescence‐positive cells over time, suggesting EV uptake by the cells. (D, E) Cells were infected with JEV followed by incubation with EVs derived from BM or AD at concentrations of 10, 20, and 30 μg for different incubation hours, and viral RNA was quantified using RT‐PCR. GAPDH mRNA level was used for normalization. (F) Mice were injected with JEV at (10^7^ pfu/ml) and treated with 50 μg of EVs through the intra‐cranial route postinfection days one and day 3. Viral RNA was quantified post 24 h of symptoms onset. All qRT‐PCR data are represented as mean ± SD; *p < 0.05, **p < 0.001.

To check the toxicity of the BM‐EVs on C17.2 cells, we treated the cells with different concentrations (10–50 μg) of EV. After 48 h post incubation, we observed >80% cell viability with 40 μg protein equivalent EVs compared to mock‐treated control cells (Figure 3A). We chose 30 μg protein equivalent EVs as the maximum concentration for subsequent study. EVs can enter cells by fusion or active uptake. To confirm that our purified EVs were competent to enter into cells, we used the membrane‐specific dye PKH67 to monitor this process. PKH67 stained the outer membrane EVs. When incubated with C17.2 cells with BM‐EVs, stained EVs were detectable in the cytoplasm within 6 h after incubation, suggesting that EVs can be internalized by C17.2 cells (Figure 3B). Additionally, flow cytometry analysis was performed to confirm the EV uptake. An increase in green fluorescence‐positive cells was observed over time, suggesting uptake of BM‐EVs by C17.2 cells (Figure 3C).

Subsequently, we analyzed whether BM‐EVs were capable of inhibiting JEV replication. C17.2 cells were infected with the JEV virus, and purified EVs have added afterward. Treatment of BM‐EVs remarkably reduced the intracellular JEV RNA level in a dose and time‐dependent manner (Figure 3D), whereas AD‐EVs showed minimal or no apparent antiviral activity (Figure 3E). Further, to test if BM‐EVs have any beneficial effect on limiting JEV viral replication in the brain, we infected the P10‐old mice with JEV through the i.c route. BM‐EVs were administered through the i.c route either 1 day or 3 days postinfection. Brains were harvested once the mice showed the symptoms. As measured by qRT‐PCR (Figure 3F), viral RNA levels were significantly reduced in mice brains when BM‐EVs were administered 3 days postinfection of the brain. Together, these results suggest the anti‐JEV effect exerted by BM‐EVs.

### 3.3 Bone marrow‐derived extracellular vesicles treatment augmented neuronal differentiation

**FIGURE 4:**
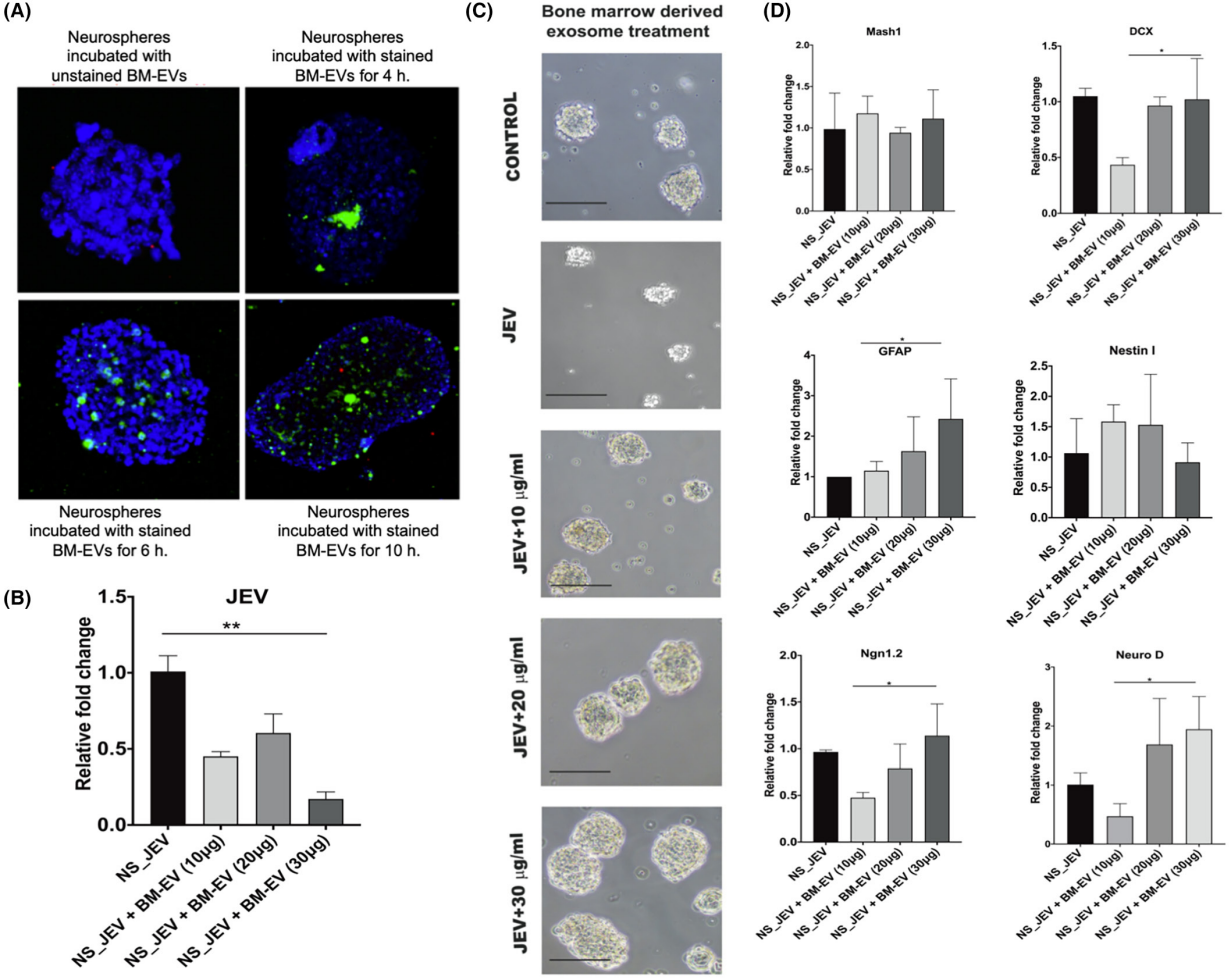
The effect of BM‐EVs on the size, growth, and functionality of the neurospheres (NS). The neurospheres (NS) were isolated from the subventricular zone of mice and cultured in vitro. The NS were infected with JEV and treated with different concentrations of EVs. (A) NS were incubated with PKH67 labeled BM‐EVs to monitor EV uptake and visualized under the confocal microscope after indicated times of incubation. (C) Spheres were visualized under the light microscope, and the sphere size and growth were checked. Significantly large and a greater number of spheres were observed in a higher dose of treatment. Scale bar = 10 μm. (B, D) Spheres were lysed to check viral RNA and various neurogenesis markers through RT‐PCR to assess the neurogenesis profile post‐BM‐EVs treatment. Significantly less viral load was observed in the higher dose group (B). The expression of DCX, GFAP, and Ngn1 is increased substantially in the treatment group (D). All qRT‐PCR data are represented as mean ± SD; *p < 0.05, ***p < 0.0005.

JEV can infect neuronal stem cells in the brain. To test the effect of BM‐EVs on JEV replication in neuronal stem cells, we purified neuronal stem cells and cultured them as free‐floating clusters of neural stem cells, called Neurospheres (NS).^32^, ^35^ After the formation of neurospheres, we first checked BM‐EVs uptake capacity by the neurospheres at different time points. As shown in Figure 4A, PKH67‐stained BM‐EVs was detectable inside the cluster within 6 h postincubation as they migrated inside the cytoplasm of the cluster. Next, we investigated the functional effect of BM‐EVs on neurospheres.

We infected the NS with JEV before incubation with different concentrations of BM‐EVs. Neurospheres were harvested 5 days after EVs incubation, and JEV RNA was detected by qRT‐PCR. Compared to PBS‐treated NS, BM‐EVs treated NS exhibited a significantly low amount of viral RNA in the treated NS (Figure 4B), confirming previous observations that BM‐EVs have anti‐JEV activity.

Compared to uninfected control (mock), the size of the JEV‐infected NS was significantly reduced, confirming the previous observation that JEV infection arrest neuronal differentiation.^32^ However, BM‐EVs treated with NS exhibited a gradual increase in size in a dose‐dependent manner, possibly indicating its role in the neuronal differentiation process (Figure 4C).

We also measured several genes that are associated with the neuronal differentiation process. Compared to uninfected control, DCX, Ngn1, and NeuroD level were significantly reduced in JEV‐infected NS, whereas no changes were observed in Mash1, GFAP, Nestin, and Ngn2 gene expression in JEV‐infected NS. Whereas in BM‐EVs treated NS, except Mash1 and Nestin, all other marker expression was increased compared to only JEV‐infected group (Figure 4D), suggesting BM‐EVs support the process of neurogenesis even in the infection scenario.

### 3.4 Bone marrow‐derived extracellular vesicles treatment delay Japanese encephalitis virus‐induced symptoms and death in mice

To understand the effect of BM‐EVs treatment on the progression of the disease, we used C57BL6 mice and infected the mice with JEV through the i.p route. After 24 h.pi, we randomly divided the infected mice into two groups. Group‐I mice (*n* = 12) received PBS, and Group‐II (*n* = 12) mice received 50 μg protein equivalent EVs twice a day up to day seven. Group‐III (*n* = 12, mock, uninfected mice) received only an equivalent volume of filtered PBS. Mice were monitored every 12 h intervals, and body weight and symptoms were noted.

**FIGURE 5:**
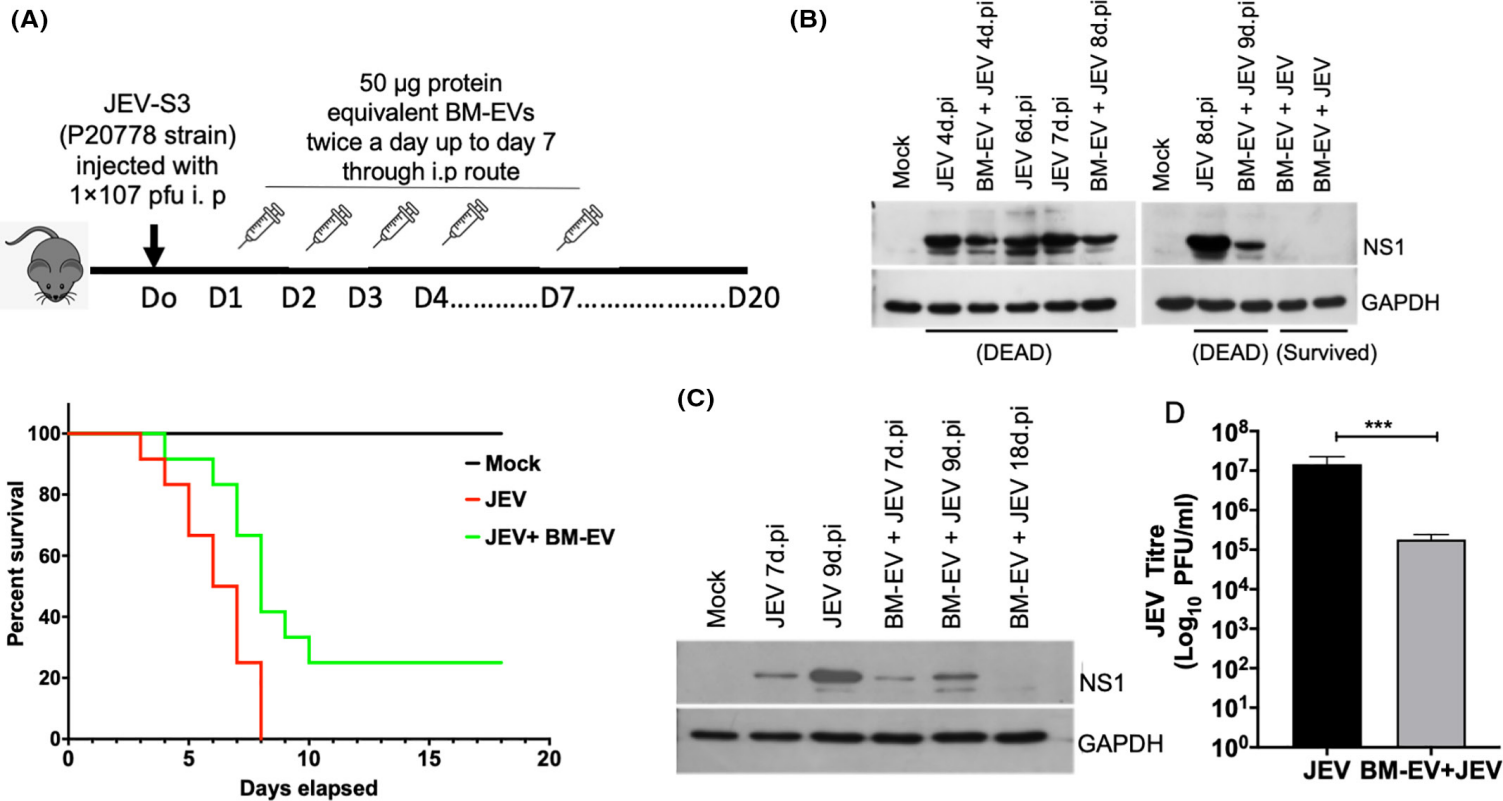
Status of virus in C57BL/6 mice post‐BM‐EVs treatment. (A) Schematic diagram of BM‐EVs treatment in JEV‐infected C57BL/6 mice. C57BL/6 mice (3–4 weeks, *n* = 12) old inoculated with JEV at (10^7^ pfu/ml) and treated with BM‐EVs at the dosage of 50 μg twice a day post 12 h of infection till day 7. The panel shows the Kaplan–Meier survival curves of animals infected and treated with BM‐EVs using a log rank. (B, C) The brains were harvested at different time points after the appearance of symptoms, and the lysate was prepared. Representative Western blots showed the expression level of JEV (NS1), and GAPDH was used as an internal control. (D) The infected brains were homogenized in MEM, and the supernatant was collected for the virus titration. The plaque assay was performed from the supernatant of both groups. The bars depicted the data from 5 subjects in each group. All qRT‐PCR data are represented as mean ± SD; ***p < 0.0005.

The JEV‐infected mice displayed reduced body weight and developed encephalitic symptoms from day‐3 onward. All JEV‐infected mice died between days 7 and 8, but the BM‐EVs‐treated mice showed delayed development of encephalitic symptoms and increased survival. Less than forty percent of the mice survived till day 8. Interestingly, 20–30% of the treated mice started developing mild encephalitis‐like symptoms postday 7 of JEV injection, but they gradually recovered and survived (Figure 5A). The survived mice were carefully monitored till day 20 postinfection for any behavioral alterations. These mice were sacrificed on day 20, and viral titer was estimated in brain tissues. Western blot was performed on the mice that died on different days and represented in Figure 5B. Although BM‐EVs treated ~70% of mice died, there is a delay in developing the symptoms. The level of viral proteins in the brain was also lower than the JEV + PBS‐treated group. We further ran another experiment where we continued BM‐EVs treatment up to day 10. We harvested mice brains from both groups at the same time point and compared the intracellular viral proteins. We observed a reduced intracellular viral protein expression in mice treated with BM‐EVs (Figure 5C). Also, we measured viral titer by plaque assay as described earlier.^28^ Overall, the Infectious viral titer was reduced significantly (~2log fold) in mice treated with BM‐EVs compared to the JEV + PBS group (Figure 5D).

**FIGURE 6:**
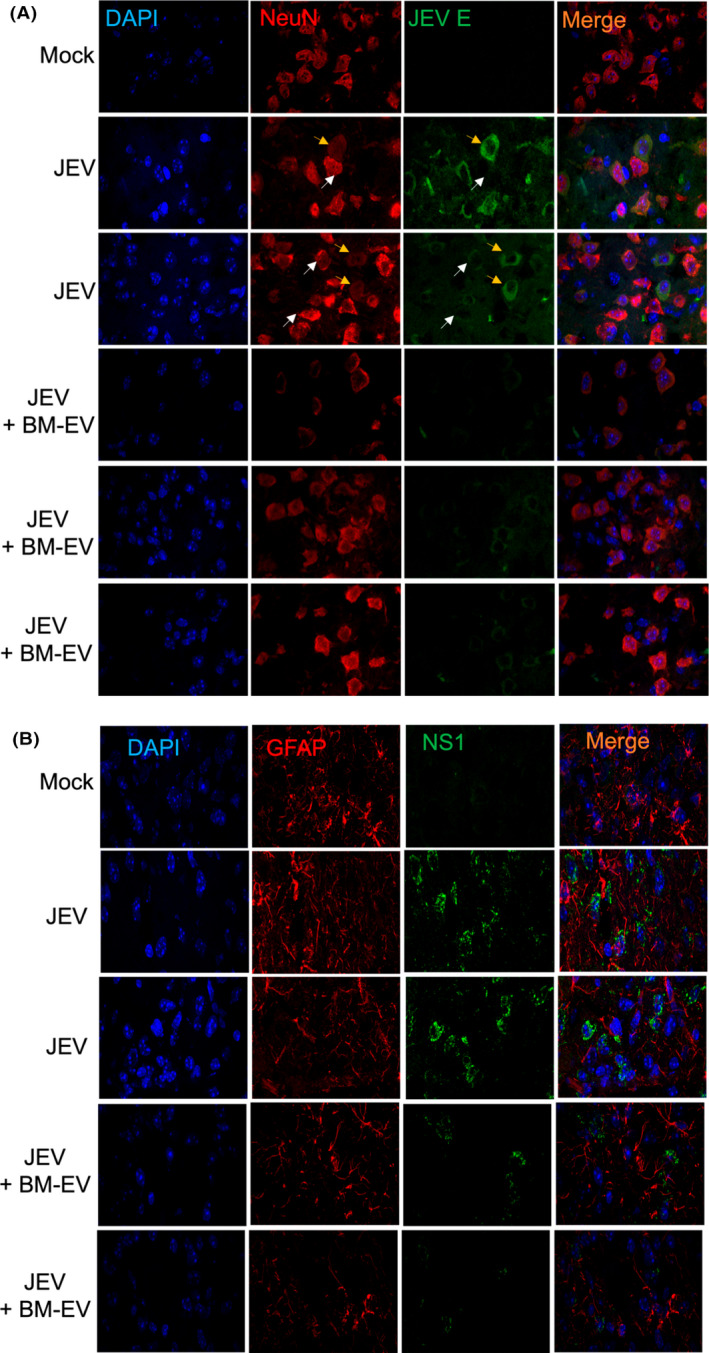
Effect of BM‐EVs treatment on neuronal infectivity and astrocyte activation upon JEV infection. C57BL/6 mice were infected with JEV (10^7^ pfu) through the intraperitoneal route followed by BM‐EVs administration. Twenty‐four‐hour postsymptoms onset brains were harvested, and sections were prepared. (A) Brain Sections from all three groups were subjected to staining for the viral expression JEV E (green), Neuron (red), and DAPI (blue) for the nucleus. Arrows (white and yellow) were used to show the inverse expression of NeuN and JEV protein. (B) Cryopreserved sections were processed for staining with the viral marker (NS1, green), Astrocyte marker (GFAP, red), and DAPI for the nucleus. All the images are captured at 60× magnification. Scale bar = 10 μm.

The viral load of JEV in the brain was further assessed by detecting E protein and NS1 expression through immunofluorescence, and the fluorescence intensity was analyzed using ImageJ (PBS *n* = 3, JEV + PBS *n* = 6, JEV + BM‐EVs *n* = 6); (three sections per mouse, five fields per section). The staining showed that BM‐EVs treatment decreased the viral load in mice brains. The neuronal‐specific nuclear protein NeuN was markedly downregulated in cells where the viral protein expression was more pronounced (marked with pointed yellow arrows), suggesting neuronal damage (Figure 6A).

Glial Fibrillary Acidic Protein (GFAP) is astrocytes' major intermediate filament protein in the CNS. Increased levels of GFAP are a hallmark feature of gliosis, a nonspecific response of astrocytes to a wide variety of inflammation and injuries in the CNS. Excess GFAP may cause or exacerbate astrocyte dysfunctions. We observed increased GFAP expression in mice brains infected with JEV only compared to mock. However, BM‐EVs treated mice exhibited suppressed expression of GFAP (Figure 6B).

**FIGURE 7:**
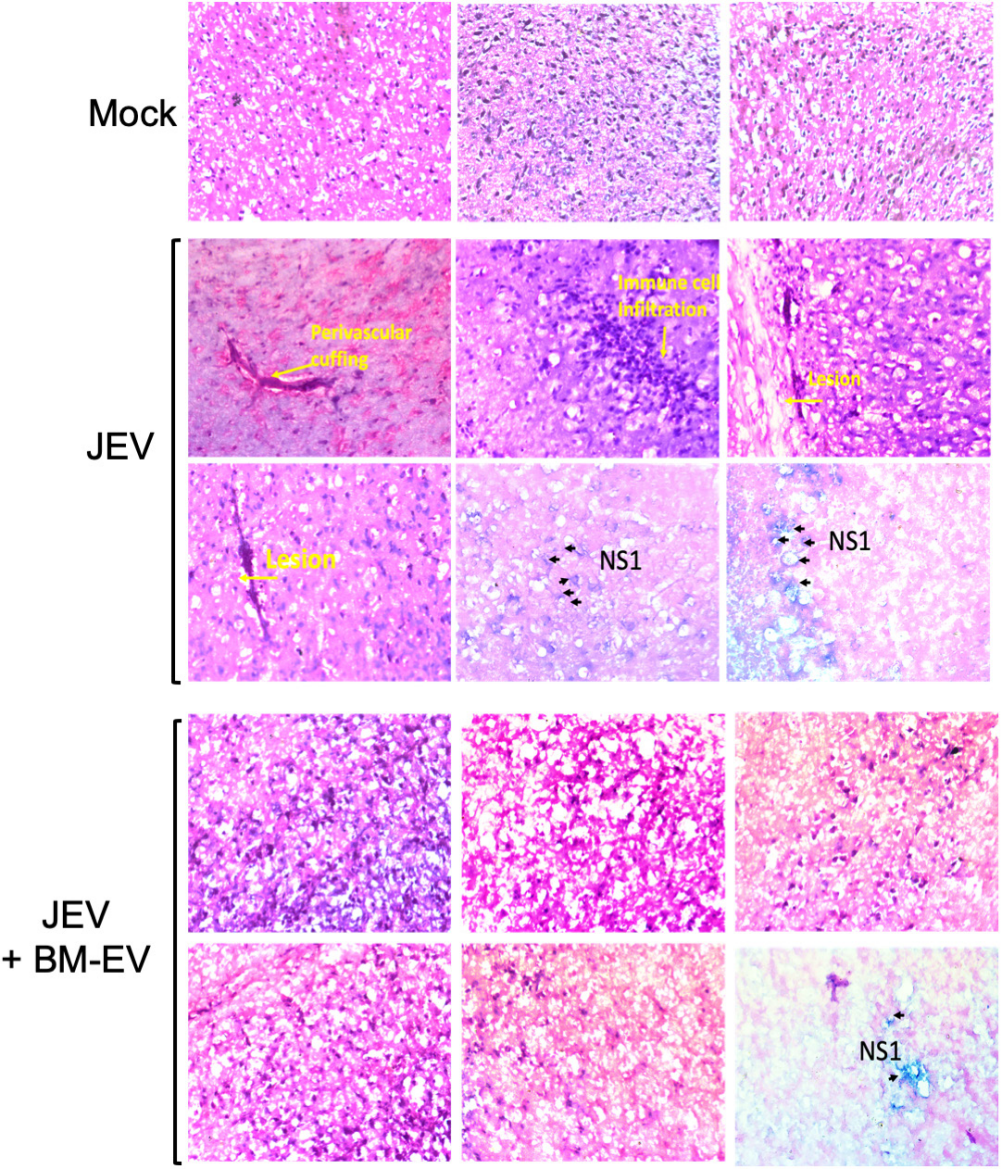
Pathological effect of JEV in C57BL/6 mice brains in mock, infected, and treated brain sections. Representative images show the staining for Hematoxylin and Eosin. The left panel in the JEV sections shows the signature of JEV infection, like perivascular cuffing, lesions, and immune cell infiltration. Comparatively, fewer lesions and cuffing were observed in BM‐EVs treated sections. The rightmost panel shows the viral expression NS1 stained with immune peroxidase counterstained with hematoxylin. Images were captured at 40× magnification.

A brain histochemistry study was also performed. Compared to the mock brain, JEV‐infected mice's brains exhibited brain lesions and increased immune cell infiltration. However, BM‐EVs showed less intense damage and fewer viral proteins compared to only JEV infected group (Figure 7).

### 3.5 Bone marrow‐derived extracellular vesicles reduce neuronal death in vivo

**FIGURE 8:**
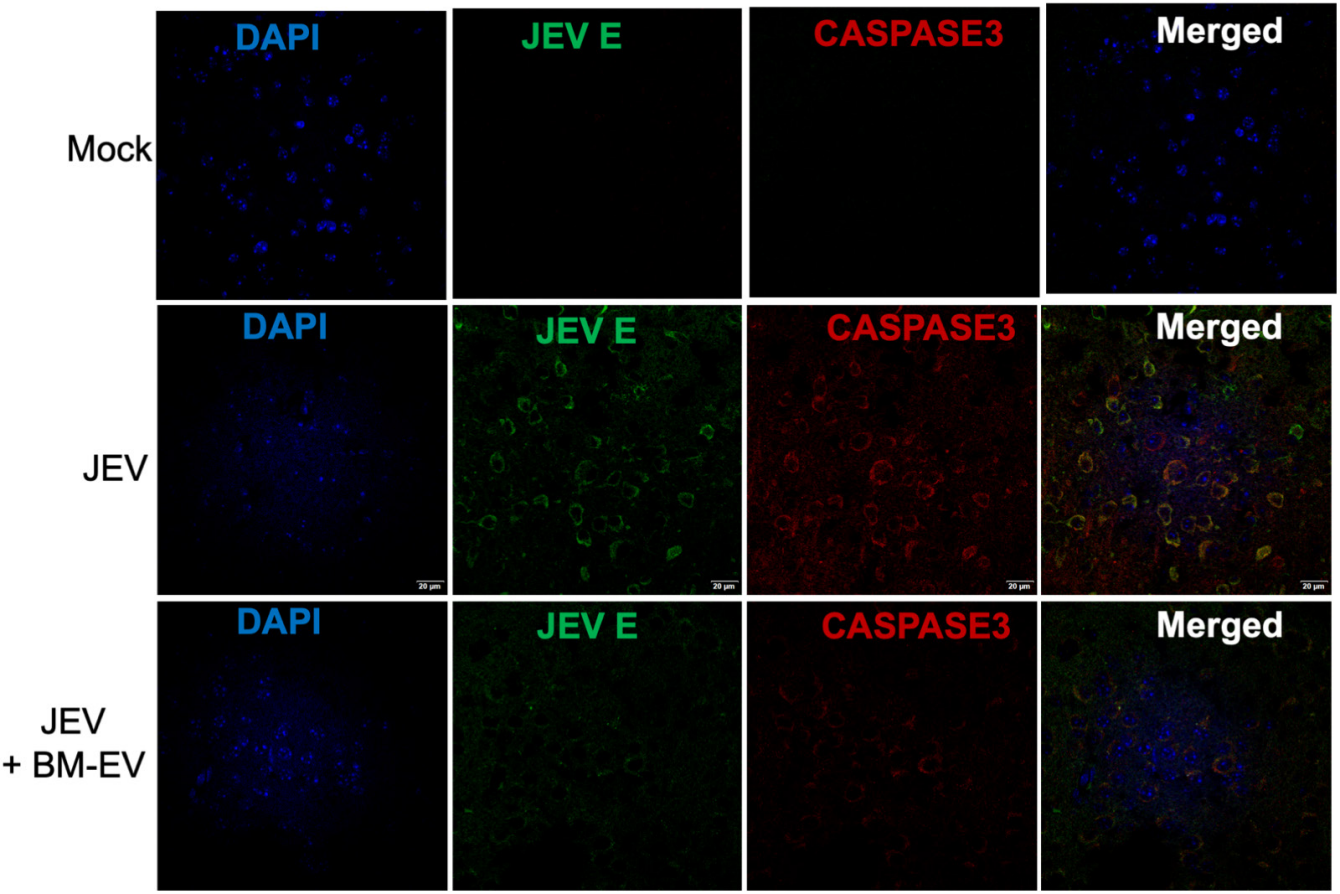
Caspase activation in JEV‐infected and BM‐EVs‐treated C57BL/6 mice brain sections. Sections were thawed and processed for viral antigen detection and caspase activation. Viral antigen was stained with JEV E antibody (Green), and caspase activation was shown using Caspase 3 antibody (red). DAPI (blue) was used to stain the nucleus. All the images were captured at 60× oil magnification. Scale bar = 20 μm.

Neuronal death is the hallmark of JEV‐induced brain inflammation and damage. The activation of caspases induces neuronal death. Caspase 3 is an effector caspase that functions as a central apoptosis regulator. It has been reported that different JEV‐infected cells, including mice and human neuroblastoma cells, trigger caspase activation and undergo apoptosis.^36^, ^37^ Therefore, we stained the mock and JEV‐infected mice brain sections for JEV E proteins and Caspase 3 and captured the images using confocal microscopy to see if BM‐EVs can alleviate neuronal damage. As compared to JEV only infected group, BM‐EVs treated mice exhibited less viral proteins and significantly reduced levels of Caspase 3 in infected mice's brains (Figure 8). The upregulation of apoptosis‐related proteins Caspase 3 was previously detected in the brains of mice infected with JEV, indicating inflammation‐related neuronal apoptosis, which becomes alleviated in BM‐EVs treatment.

### 3.6 Bone marrow‐derived extracellular vesicles treatment augmented interferon stimulating genes (ISGs) and attenuated TNFα and IL6 expression

**FIGURE 9:**
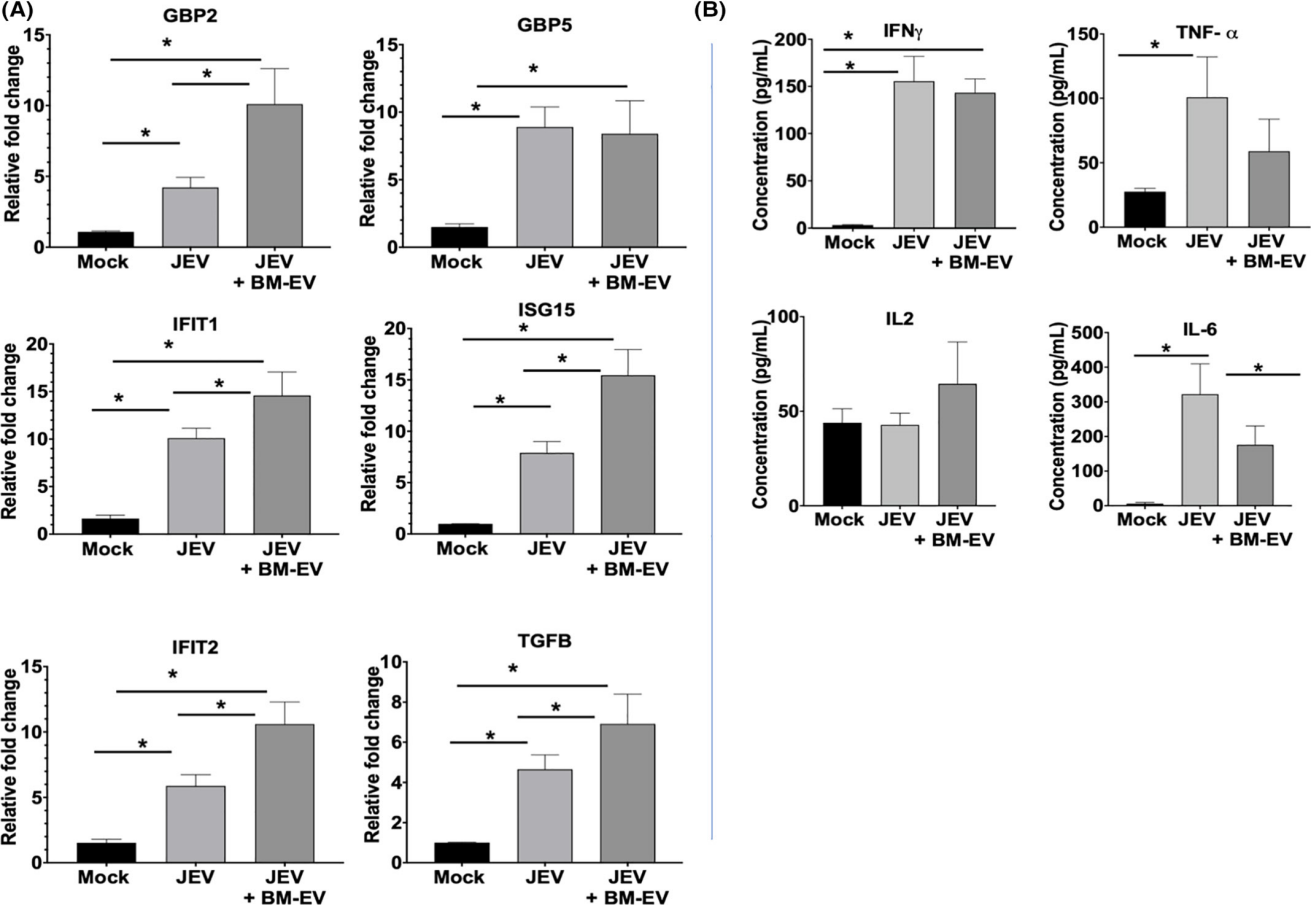
Expression pattern of interferon‐stimulated genes and cytokines in JEV‐infected and BM‐EVs treated mice brain. (A) C57BL/6 mice were inoculated with JEV (10^7^ pfu) and treated with 50 μg of EV protein twice a day for 7 days. Brains were harvested post symptoms severity and processed for RNA isolation and protein lysate preparation. ISGs expression was quantified using RT‐PCR by using GAPDH as an internal control. (B) The cytokine expression pattern was checked by flow cytometry using a cytokine bead array kit. The data were analyzed using FCAP and qognit software. All qRT‐PCR and protein data are represented as mean ± SD; *p < 0.05.

Interferon‐stimulated genes (ISGs) play an essential role in antiviral response. Therefore, we measured ISGs gene expression in the JEV‐ infected and ‐uninfected brain using the RT‐PCR method. The ISGs, for example, GBP2, TGFß, IFIT1, IFIT2, and ISG15 levels, were significantly enhanced in BM‐EVs treated mice compared to only JEV‐infected mice (Figure 9A). Simultaneously, a Cytokine bead array was performed in brain whole lysates with or without treated with BM‐EVs. Increased TNFα and IL6 is the hallmark of JEV infection in the brain. IFNγ levels were found to significantly increase in JEV only and JEV + BM‐EVs group compared to mock. No difference in IFNγ levels was observed in mice with or without BM‐EVs (Figure 9B). Whereas IL‐2 level was significantly high in BM‐EVs treated mice. IL‐2 stimulates regulatory T cells, whose role is to control inflammation. In contrast, The proinflammatory cytokines, for example, IL6 and TNFα levels, were reduced significantly in BM‐EVs treated mice, suggesting that BM‐EVs can reduce pro‐inflammatory cytokines like TNFα and IL6 (Figure 9B).

### 3.7 Bone marrow‐derived extracellular vesicles treatment modulates the abundance of infiltrating immune cells in infected mice

**FIGURE 10:**
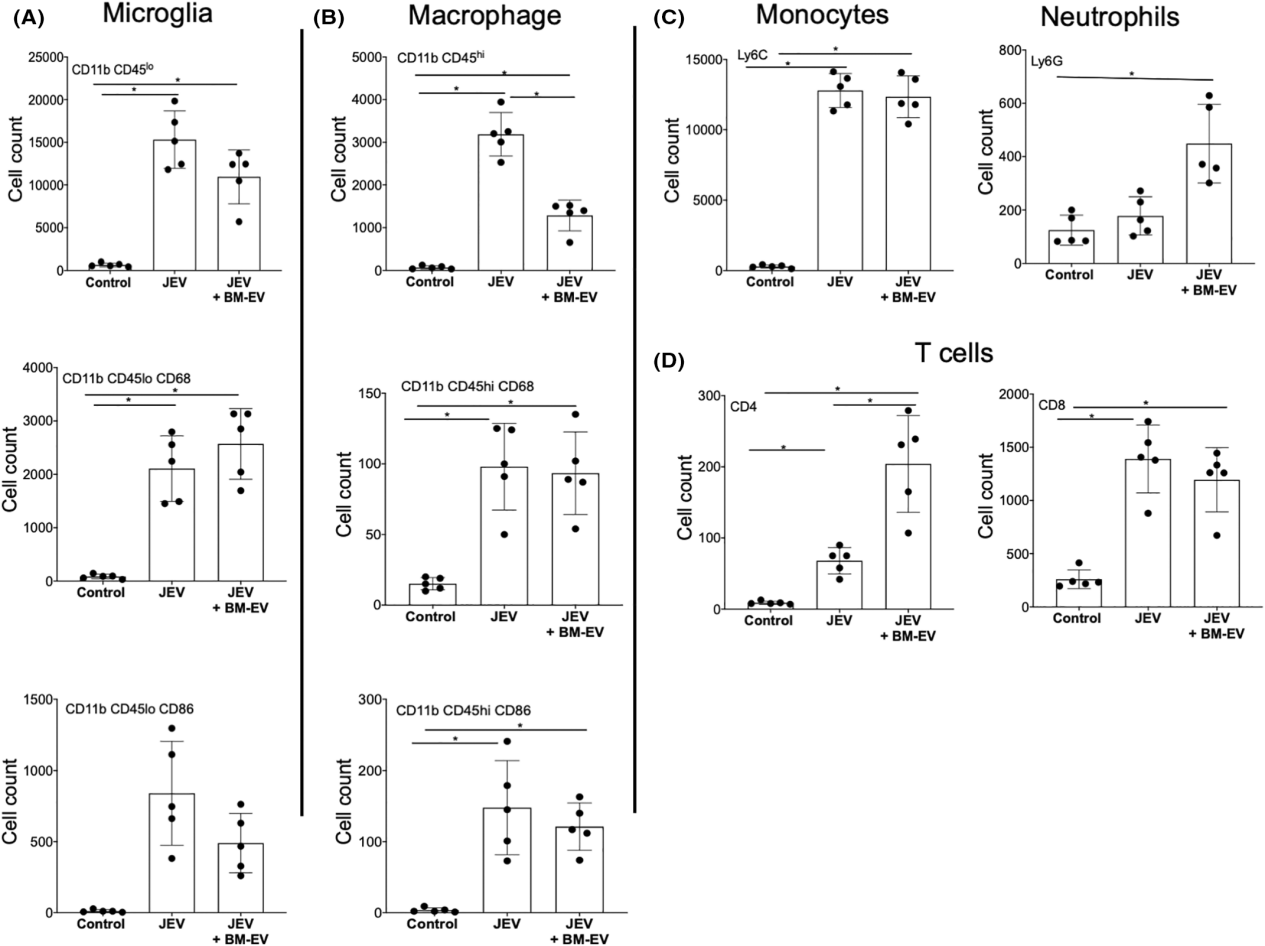
Infiltrating immune cells profile of JEV‐infected and treatment group 24 h post symptoms onset. C57BL/6 (*n* = 5) were infected with JEV, followed by treatment. Brains were harvested 24 h postsymptom onset, and the sample was prepared for FACS acquisition. (A, B) Cells were stained for microglia and macrophages markers and categorized as per the expression pattern of cell surface markers CD11b, CD86, and CD45. (C) Expression pattern of monocytes and neutrophils was checked by staining them with Ly6C and Ly6G. (D) Bar graphs showed the expression pattern of the T cell population. Each dot represents cells from individual mice. *p < 0.005.

JEV infection is associated with increased blood–brain barrier disruption leading to infiltration of peripheral immune cells. MSCs can modulate peripheral immune cells. To check if EVs can modulate the abundance of immune cells in brains, we investigated the status of infiltrated cells using FACS. The mice brains were harvested upon the development of the symptoms, and cells were stained with respective antibodies representing markers for microglia, macrophages, monocytes, neutrophils, and T cells. Activated primary microglia differ from other blood macrophages in the expression levels of markers like CD11b/CD45low/high, CD68, and CD86. As compared to microglial cells (CD11bCD45low), a reduced number of macrophages (CD11bCD45high) were observed in JEV+ BM‐EVs treated group in comparison to only JEV‐infected mice (Figure 10A,B). However, we did not observe significant differences in CD68 and CD86 expression between JEV only and the JEV + BM‐EVs treated group (Figure 10A,B). JEV infection increases infiltrated monocyte (Ly6C+) numbers in the infected brains compared to mock. However, BM‐EVs treatment does not affect Ly6C+ cell abundance in the JEV‐infected mice brains (Figure 10C).

On the contrary, BM‐EVs treatment significantly increases neutrophil infiltration in the JEV‐infected mice brain. JEV infection increases CD4+ T cells in the mouse brain compared to mock. However, BM‐EVs treatment further significantly increases CD4+ T cells in the brain (Figure 10D). However, no difference in CD8+ T cells was observed in JEV only and JEV+ BM‐EVs group, suggesting that BM‐EVs treatment can modulate the abundance of the peripheral immune cells in the brain.

## 4 DISCUSSION

MSC‐derived EVs have recently gained importance as cell‐free therapeutics modalities in many neurological and infectious diseases. It was reported that MSCs‐EVs promoted neurological recovery via regulating neuroinflammation and secreting neurotrophic factors. Here, we undertook a comprehensive study to look into the effect of BM‐EVs on JEV replication and pathogenesis. To delineate the outcome, we utilized both in vivo model of JEV infection and in vitro neurospheres culture to study progressive JEV infection and demonstrated the effect of BM‐EVs on virus replication, survival, Interferon response and cytokine expression, neuronal death, and immune cell infiltration.

It is reported earlier that MSCs cell transplantation in vivo mice model lessens JEV pathogenesis.^10^ MSC‐EVs also retain parental cell properties. BM‐EVs' neuroprotective role was evident from the rescued neurosphere size and decreased cell death shown by reduced expression of caspase3. Neurospheres, are small clusters of nerve stem cells grown in the laboratory and widely used to study neurogenesis. It was previously reported by Das and Basu^35^ that the ability of JEV‐infected subventricular zone cells to form neurospheres is severely compromised. JEV infection in neuronal progenitor cells suppresses the cycling ability of these cells, preventing their proliferation.

In our study, BM‐EVs treatment in JEV‐infected neurospheres significantly reduced viral RNA, and increased levels of different gene markers were also observed. Further, JEV infection induces Caspase activation in the brain leading to neuronal death. We observed reduced caspase3 activation in BM‐EVs treated infected mice brains, supporting its neuroprotective role. Like in vitro studies, BM‐EVs enhanced survivability in mice models of infection and effectively reduced viral load. However, instead of reduced viral load, about 70% BM‐EVs treated mice eventually died, suggesting other factors probably overpowering the beneficial effect of BM‐EVs. The infectious virus particles, viral proteins, and immune cell infiltrations are evident in BM‐EVs treated mice brains. These further suggest that BM‐EVs treatment does not protect BBB against lethal doses of JEV infection. It is to be noted that infiltration of peripheral immune cells may shape the outcome of the immune response in the brain. Our study did not observe much difference in microglial activation status and monocyte number. However, fewer macrophage populations were observed in BM‐EVs treated mice.

On the other hand, a significant increase in CD4+ T‐cells and neutrophils population was observed in BM‐EVs treated mice. However, the CD8+ populations in JEV only and BM‐EVs treated groups remain comparable. These results suggest that BM‐EVs preferentially enhance infiltration of CD4+ and neutrophil population over the monocytes and macrophages. Increased preferential infiltration of neutrophils in BM‐EVs treated mice may have significance in brain inflammation. In the brain, microglia promote neutrophils to secrete more pro‐inflammatory cytokines and initiate neurodegeneration. It has been reported in multiple studies that elimination of neutrophils or inhibition of neutrophil recruitment shows some protective effects in brain injury.^38^, ^39^, ^40^ Thus, increased neutrophil infiltration in the brain could be the cause of death observed in BM‐EVs treated mice.

Immune regulation is considered critical to disease progression. A study showed that the infiltrated neutrophils in the central nervous system suppress T cell responses, and neutrophil suppressor activity depended on IFNγ production by target T cells.^41^ In our study, we observed increased CD4 T and neutrophils along with increased IFNγ in the brain. However, we did not check the Treg population. Further study may warrant understanding the role of neutrophils on T cell function and inflammation.

The overproduction of inflammatory cytokines is the leading cause of neuropathology during JE. Thus, inhibiting the inflammation during the progression of JE has been shown to reduce neuronal death and promote neurogenesis. An appropriate immune and inflammatory response during the early phase of infection is critical for clearance of the virus and regeneration of the nervous system. However, the overproduction of IFNγ and TNFα accelerated the neuroinflammation. Thus, it is crucial to balance immune and inflammatory responses for JE treatment. For BM‐EVs treated mice, although we observed reduced expression of inflammatory cytokines TNFα and IL6, enhanced expression of IFNγ and IL2 and different ISGs were observed. Increased IFNγ and IL2 also result in increased infiltrations of immune cells in the brain (Figure 10).

**FIGURE 11:**
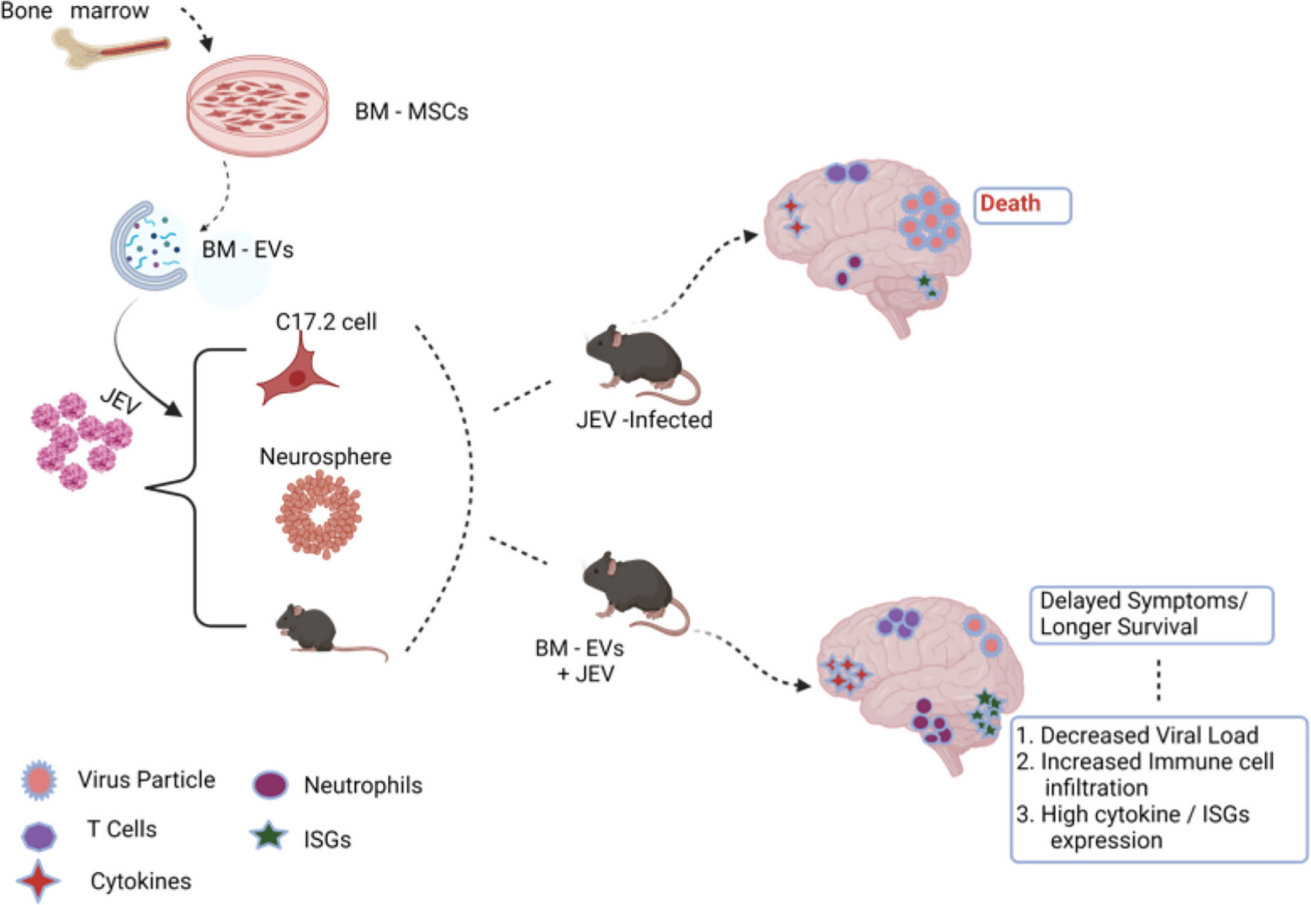
Schematic diagram representing the effect of BM‐EVs treatment on Japanese Encephalitis virus replication and immune response.

Overall, our data confirm the antiviral and immunomodulatory role of BM‐EVs in JEV infection (Figure 11). BM‐EVs seem to alter immune cell infiltration into the treated brain and alleviate JEV‐induced inflammation and fatality in mice. Further study is warranted to understand the role of the increased neutrophil population in brain inflammation and disease outcome.

## AUTHOR CONTRIBUTIONS

S.M., A.B, and Ar.B. conceived the study, secured the funding, and designed the experiments. N.S. and A.T. performed the in vitro and animal experiments. S.G. performed stem cell culture, exosome isolation, and characterization, while S.M. provided guidance. Sr.M. and N.S. Performed neurospheres isolation, culture, and related assays. N.S., A.T, S.G., and S.M. were involved in data analysis. S.M., A.B., and Ar.B. wrote the manuscript, and all authors contributed to editing the document. All authors have read and agreed to the published version of the manuscript.

## CONFLICTS OF INTEREST

The authors have no relevant financial or nonfinancial interests to disclose.

## CONSENT TO PUBLISH

All authors have given their consent to publish the manuscript.
